# Supplementary material for: Synthesis, Characterization, X-ray Molecular Structure, Antioxidant, Antifungal, and Allelopathic Activity of a New Isonicotinate-Derived meso-Tetraarylporphyrin
Source: Molecules. 2024 Jul 3;29(13):3163. doi: 10.3390/molecules29133163 (PMC11243641; doi:10.3390/molecules29133163)
Supplement: Supplementary file 1 [file molecules-29-03163-s001.zip › molecules-3045345-supplementary.pdf]

Molecules
Supplementary Material:

Electronic Supporting Information for

Synthesis, Characterization, X-ray Molecular Structure, Antioxidant, Antifungal and Allelopathic Activity of a new Isonicotinate-derived *meso*-Tetraarylporphyrin

Nour Elhouda Dardouri [1], Soukhayna Hrichi [1,2], Pol Torres [3], Raja Chaâbane-Banaoues [2], Alessandro Sorrenti [3], Thierry Roisnel [4], Ilona Turowska-Tyrk [5], Hamouda Baba [2], Joaquim Crusats [3,6], Albert Moyano [3,*], and Habib Nasri [2,*]

1 University of Monastir, Laboratory of Physical Chemistry of Materials (LR01ES19), Faculty of Science of Monastir, Avenue de l'Environment, 5019 Monastir, Tunisia
2 University of Monastir, Faculty of Pharmacy, Laboratory of Medical and Molecular Parasitology-Mycology (LP3M), LR12ES08, 5000, Monastir, Tunisia
3 Section of Organic Chemistry, Department of Inorganic and Organic Chemistry, Faculty of Chemistry, University of Barcelona, C. de Martí i Franquès 1-11, 08028 Barcelona, Spain
4 Institute of Chemical Sciences of Rennes, UMR 6226, University of Rennes 1, Beaulieu Campus, 35042 Rennes, France
5 Faculty of Chemistry, Wrocław University of Science and Technology, Wybrzeże Wyspiańskiego 27, 50-370 Wrocław, Poland
6 Institute of Cosmos Science, University of Barcelona, C. de Martí i Franquès 1-11, 08028 Barcelona, Spain

SUMMARY

- Spectroscopic data for aldehyde **4** (ESI2-ESI3)
- Spectroscopic data for porphyrin **1** (ESI3-ESI5)
- Crystal structure description of porphyrin **1** (ESI6-ESI10)

Spectroscopic data for aldehyde **4**

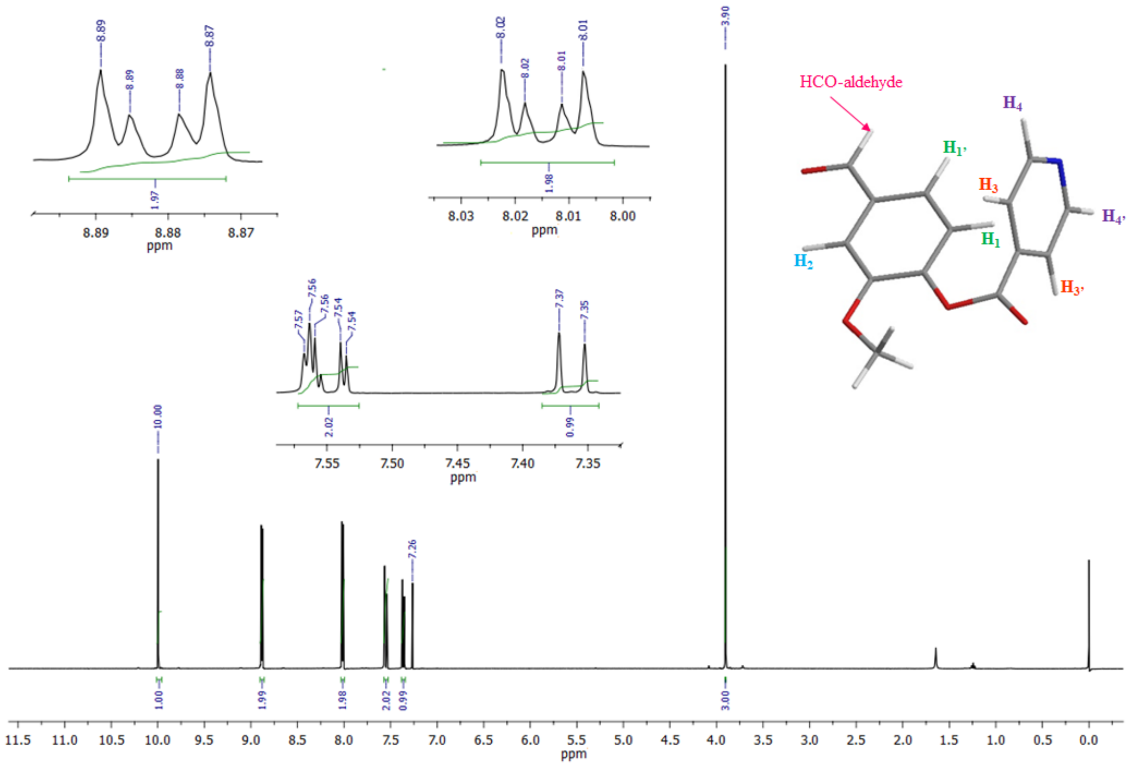

Figure S1. 400 MHz ^{1}H NMR spectrum of 4-formyl-[2-methoxy]phenyl isonicotinate **4**, recorded in $CDCl_3$. The concentration is ~ 10^{-3} M.

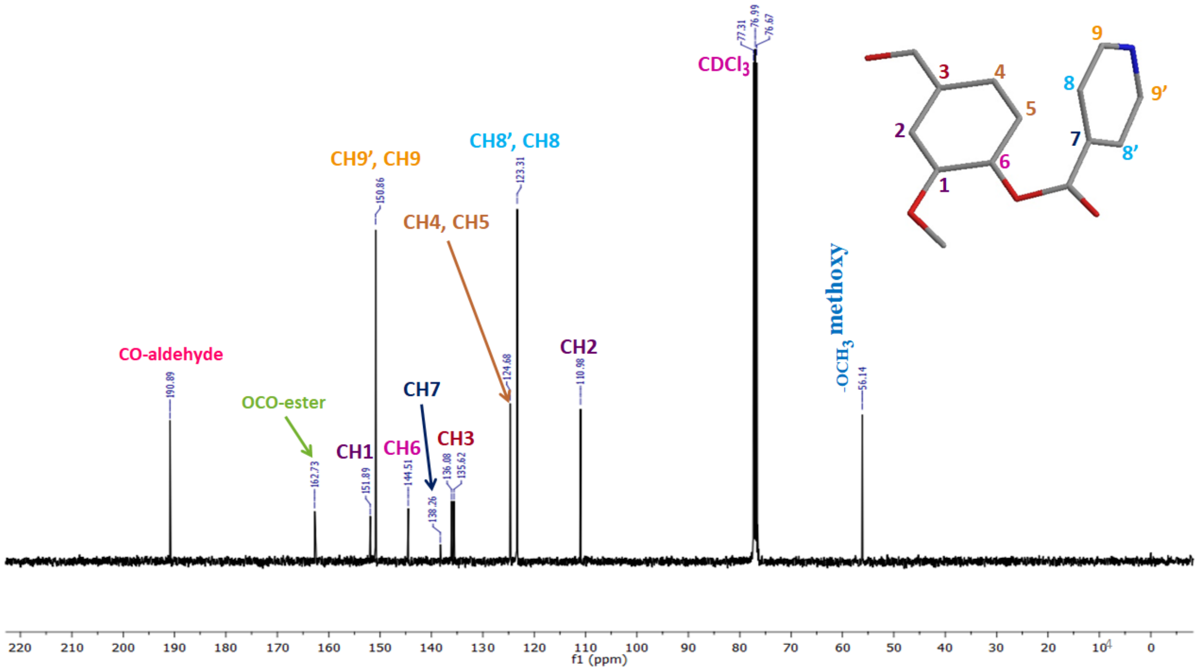

Figure S2. 151 MHz ^{13}C NMR spectrum of 4-formyl-[2-methoxy]phenyl isonicotinate **4**, recorded in $CDCl_3$. The concentration is ~ 10^{-3} M.

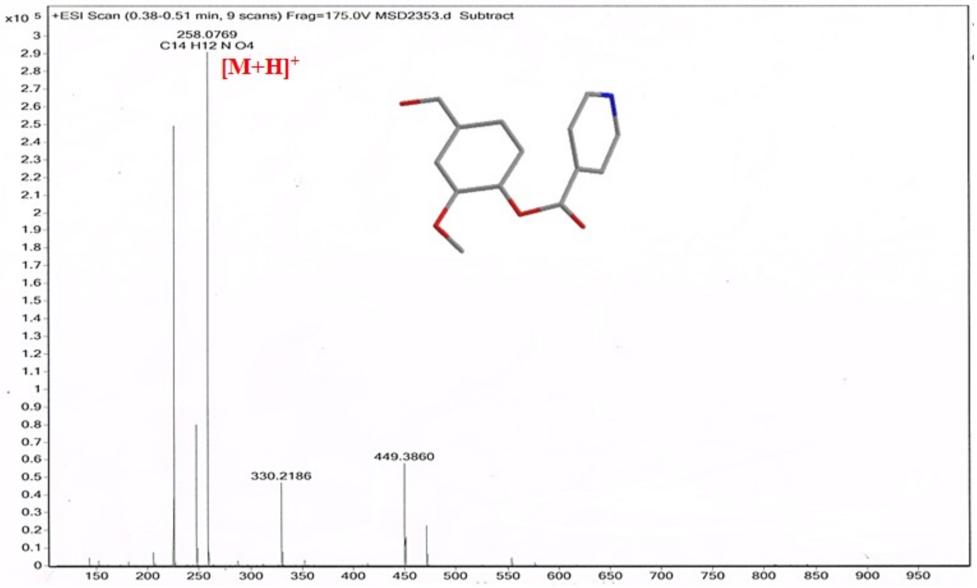

Figure S3. ESI-HRMS spectrogram of 4-formyl-[2-methoxy]phenyl isonicotinate **4**.

Spectroscopic data for porphyrin **1**

The ^{1}H and ^{13}C NMR spectra of compound **1** were recorded in deuterated chloroform at 298 K (Figures S4 and S5). Figure S4 shows characteristic inner pyrrole proton signals at -2.77 ppm. The β-pyrrole protons resonate as a singlet at 9.00 ppm, the protons of the phenyl groups and the pyridyl groups of the H_2TMIPP porphyrin resonate in the rage 8.9 – 7.5 ppm while the methoxy protons resonate at 3.95 ppm. The ^{13}C NMR spectrum of1**1** (Figure S5) shows, inter alia, signals at 56.23 and 193.31 ppm corresponding to the -C=O and the OMe groups of the H_2TMIPP porphyrin.

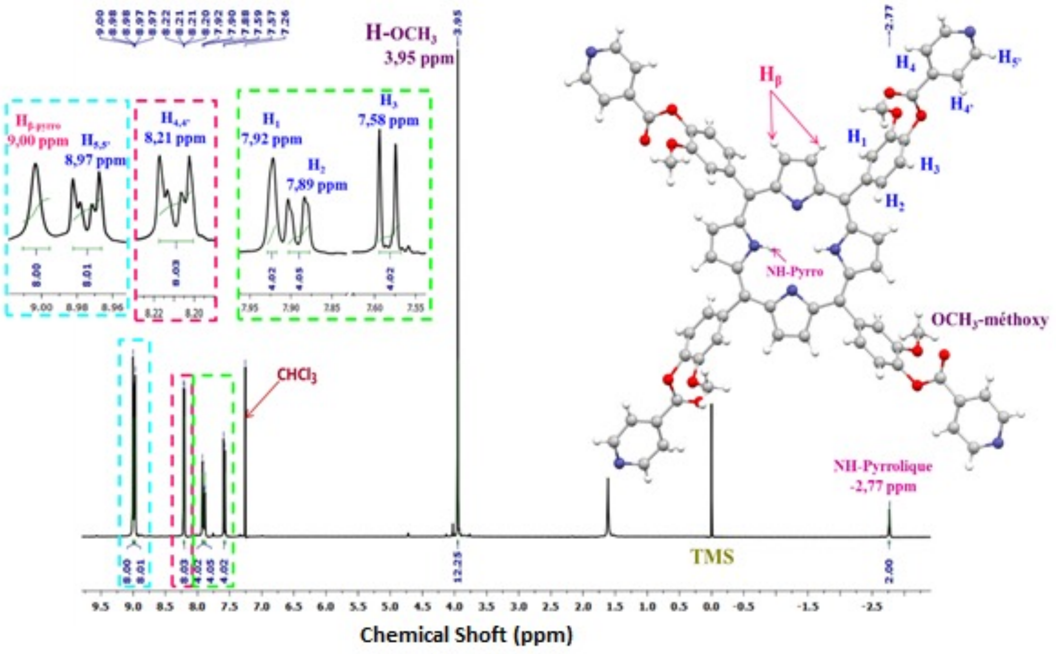

Figure S4. ^{1}H NMR spectrum (400 MHz) of the free base porphyrin H_2TMIPP recorded in $CDCl_3$ with a concentration of ~ 10^{-3} M.

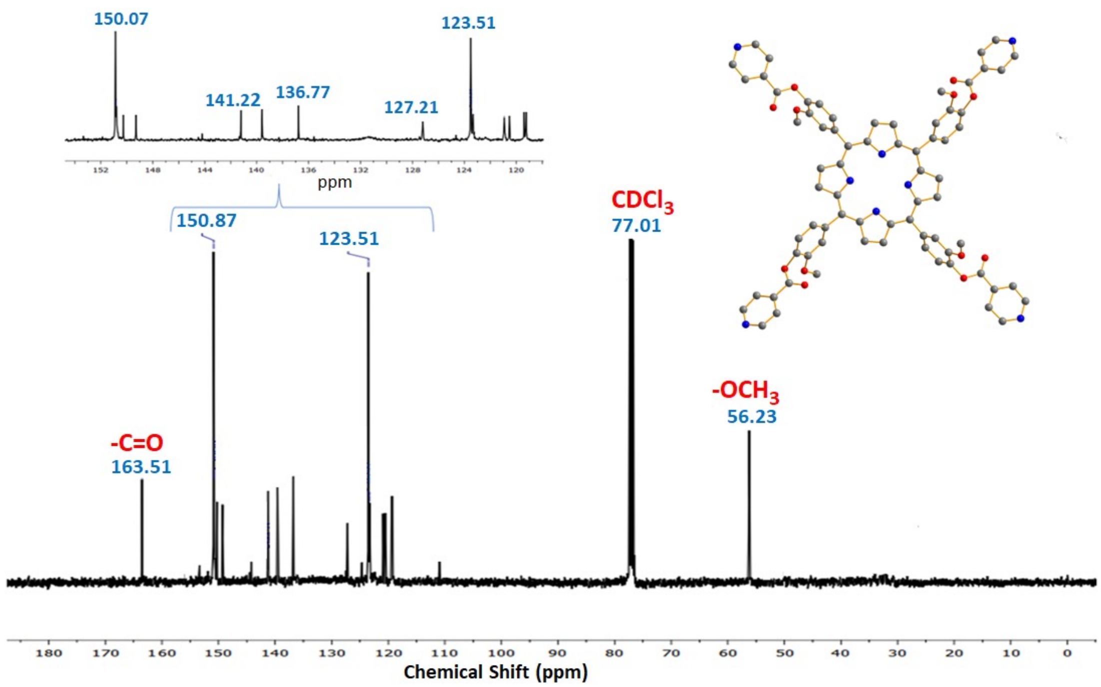

Figure S5. ^{13}C NMR spectrum (151 MHz) of the free base porphyrin H_2TMIPP recorded in $CDCl_3$ with a concentration of ~ 10^{-3} M.

The IR spectrum of H_2TMIPP (**1**) is depicted in Figure S6. The absorption band corresponding to the ν(N-H) stretching frequency of the pyrrole rings appears at 3315 cm^{-1}. The C-H bonds exhibit stretching vibrations ν(C-H) as a small singlet corresponding to the pyridyl groups of the H_2TMIPP porphyrin at 3055 cm^{-1} and as a triplet corresponding to the phenyl groups of the porphyrin between 2956 and 2852 cm^{-1}. The bending vibration δ(CCH) of the H_2TMIPP porphyrin appears as a medium absorption band at 978 cm^{-1} which is slightly high field shifted compared to the related *meso*-tetra(*para*-methoxyphenyl)porphyrin (H_2TMIPP), which appears at about 965 cm^{-1}.

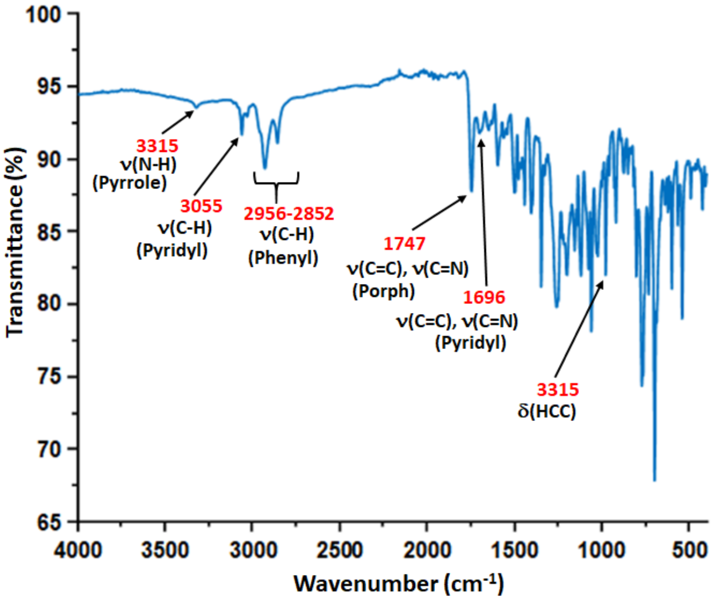

Figure S6. Neat IR spectrum of H_2TMIPP (**1**).

The experimental variation of $(\alpha h\nu)^2$ versus the photo energy ($E = h\nu$) of compound **1** is illustrated in Figure S7, where α is the absorption coefficient, h is the Planck constant, and ν is the frequency ($\nu = 1/\lambda$). The Tauc plot method (see ref. [30] in main text) was used to determine the optical gap Energy (Eg-op) which is 1.881 eV.

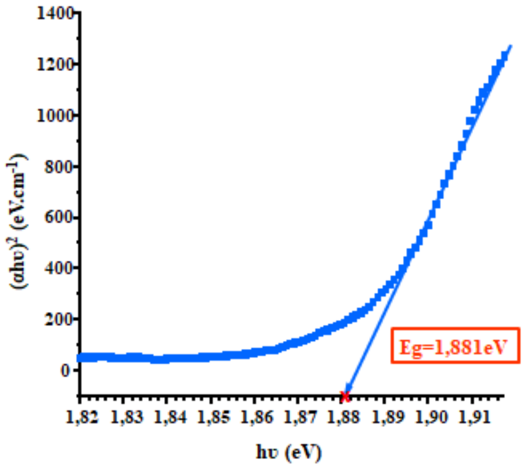

Figure S7. Plots of $(\alpha h\upsilon)^2$ versus the photon energy ($h\nu$) of H_2TMIPP. hν is the incident photon energy and α is the absorption coefficient.

Figure S8 represents the ESI-HRMS mass spectrum solution ($CHCl_3$) of compound **1** in positive ion mode, which shows the $[H_2TMIPP{+}H]^+$, $[H_2TMIPP{+}2H]^{2+}$, $[H_2TMIPP{+}3H]^{3+}$ and $[H_2TMIPP{+}4H]^{4+}$ fragments with experimental and theoretical *m/z* values of 1219.3617/1219.3621, 1220.3647/1219.3633, 1221.3674/1221.3644 and 1222.3676/1222.3652, respectively.

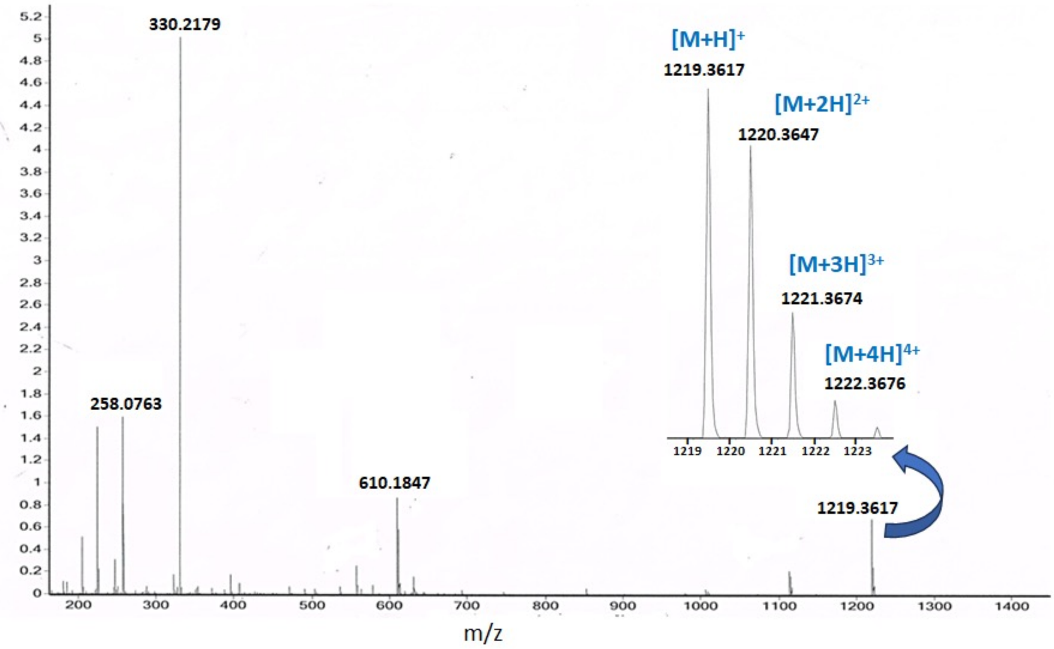

Figure S8. ESI-HRMS full spectrogram of H_2TMIPP. The insets show enlarged views. The solvent used is chloroform, with a concentration of 5.10^{-3} M.

Crystal structure description of porphyrin **1**

X-ray quality crystals of compound **1** were obtained by slow diffusion of the *n*-hexane non-solvent through a concentrated chloroform solution containing our free base *meso*-arylporphyrin. Compound **1** crystallizes in the monoclinic crystal system with the $P2_1/c$ space group and the asymmetric units contains half H_2TMIPP molecule and one half *n*-hexane non-solvent molecule leading to the formula: $H_2TMIPP^{\bullet}C_6H_{14}$ (**1**). The crystallographic data and structural refinement details of **1** are shown in Table S1.

Table S1. Crystal data and structural refinement for the *meso*-tetrakis{4-(3-methoxy)phenyl isonicotinate}porphyrin *n*-hexane monosolvate with the formula : $H_2TMIPP^{\bullet}C_6H_{14}$ (**1**).

Formula	$C_{78}H_{64}N_8O_{14}$
M.W.	1305.37
Crystal System	monoclinic
Crystal	$P2_1/c$
a (Å)	14.9807(16)
b (Å)	14.9439(16)
c (Å)	14.9053(15)
α (°)	90
β (°)	92.393(4)
γ (°)	90
V (Å^3)	3333.9(6)
Z	2
$\rho_{calc.}$/ g.cm^{-3}	1.300
μ/ mm^{-1}	0.089
F(000)	*1368*
Crystal size (mm^3)	0.760×0.200×0.130
Crystal Color	dark blue
Crystal Shape	prism
T(K)	150 (2)
θ_{min} - θ_{max} (°)	1.926-25.000
Limiting indices	$-17 \leq h \leq 17$, $-17 \leq k \leq 16$, $-17 \leq l \leq 17$
R(int)	0.0596
Total/Unique data	22628/5858
Observed data [$F_o > 4\sigma(F_o)$]	4149
Parameters/Rest	676/1307
S [Goodness of fit]	1.103
R_1 [a], wR_2 [b] [$F_o > 4\sigma(F_o)$]	R_1 = 0,1102; wR_2 = 0,2582
R_1 [a],wR_2 [b] [all data]	R_1 = 0,1434; wR_2 = 0,2811
Min./max. res. (eÅ^{-3})	0.327 /-0.252
CCDC	2309201

[a] : $R1 = \Sigma||Fo|-|Fc||/\Sigma|Fo|$, [b] : $wR2 = \{\Sigma[w(|Fo|^2-|Fc|^2)^2]/\Sigma[w(|Fo|^2)^2]\}^{1/2}$.

An ORTEP diagram of the H_2TMIPP free base porphyrin is shown in Figure S9.

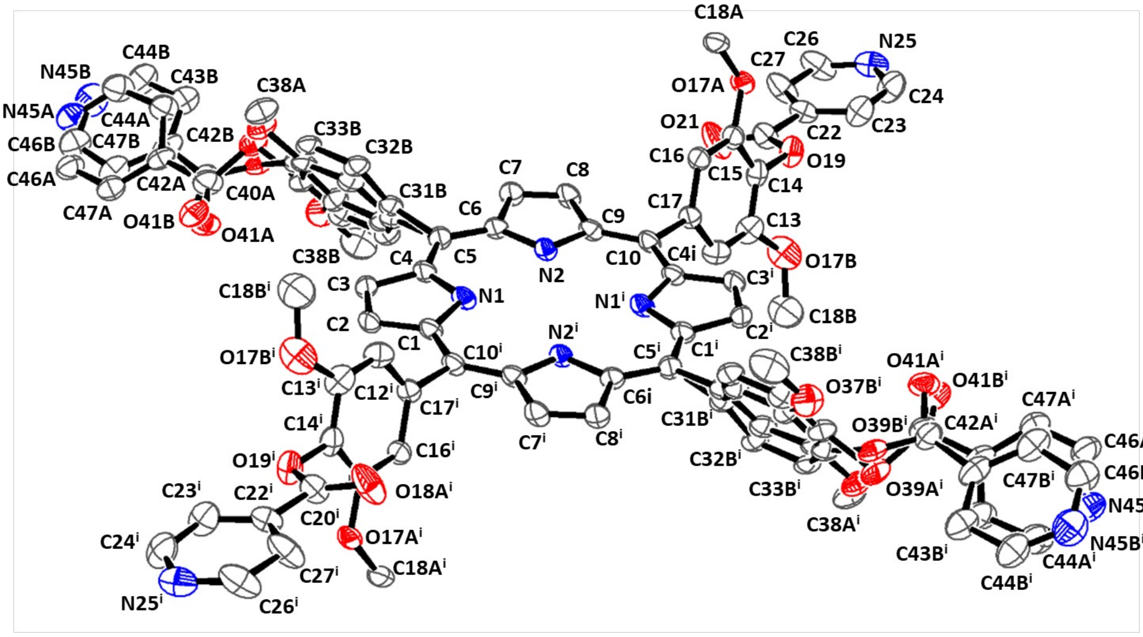

Figure S9. Ortep diagram of the free base porphyrin H_2TMIPP **1**, displaying the atom-labeling scheme. Labels with " i " correspond to the inversion center symmetry related atoms The ellipsoids are drawn at the 30% probability level and the hydrogen atoms are removed for clarity.

A selection of distances and angles of compound **1** is given in Table S2.

Table S2. Selected bond lengths (Å) and angles (deg) for H_2TMIPP (**1**).

N1-C4	1.378(6)	C4-N1-C1	106.5(4)
N1-C1	1.379(5)	C6-N2-C9	108.9(4
N2-C6	1.362(6)	N1-C1-C10	125.3(4)
N2-C9	1.362(6)	N1-C1-C2	109.4(4)
O17A-C18A	1.415(7)	C10-C1-C	2125.3(4)
C14-O19	1.409(5)		

A part of the crystal packing of this porphyrinic species is illustrated by Figure S10.

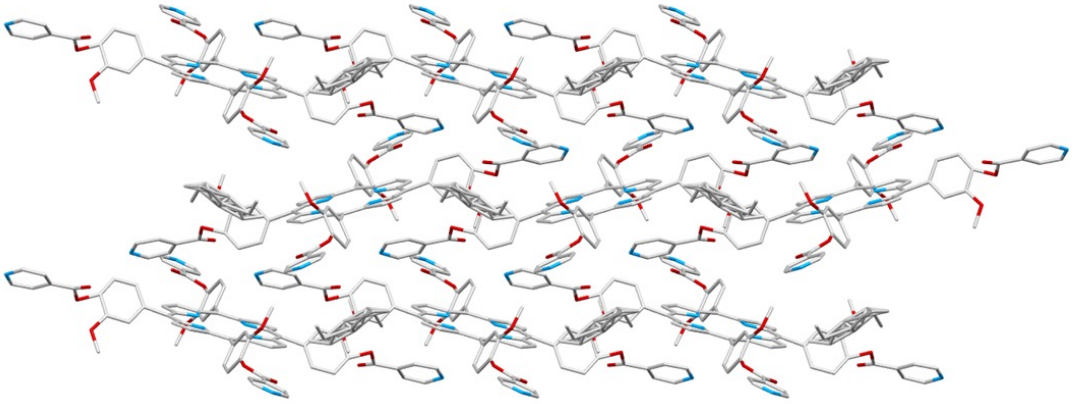

Figure S10. View of the crystal lattice of compound **1**·(*n*-hexane) view along the [001] direction.

When the disordered *n*-hexane solvent molecule is removed from the crystal lattice of **1**, voids representing 13.5% of the unit cell and a volume of 451,5 Å^3 appear which are parallel to the [001] direction (Figure S11).

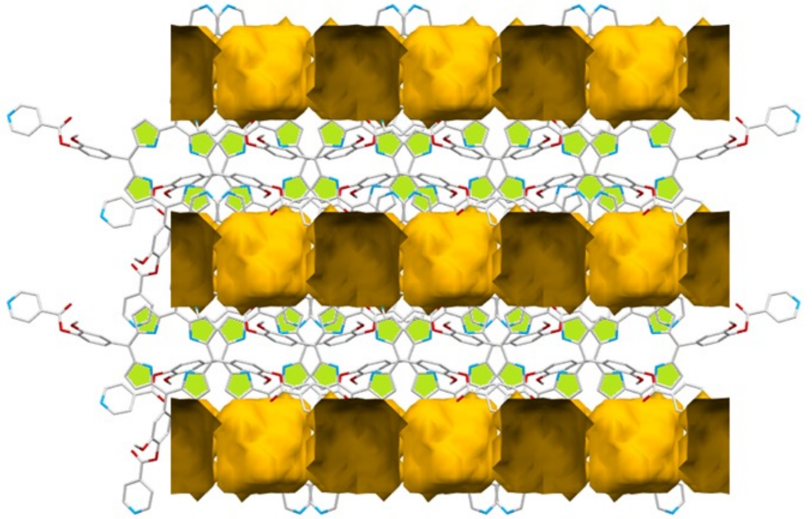

Figure S11. Packing diagram of complex **1** after removing the *n*-hexane non-solvent molecules showing voids down the [001] direction. A ball radius of 1.2 Å and a grid of 0.7 Å were used to calculate the voids.

As shown in Figure S12, two H_2TMIPP molecules are linked together by weak intermolecular interactions types C—H···O, C—H···N and C—H···Cg where Cg is the centroid of a pyrrole ring (Table S3). The crystal lattice of compound **1** is further consolidated by Cg…Cg π intermolecular interactions involving the centroids Cg1, Cg2, Cg3 and Cg4 where Cg1 is the centroid of the pyrrole ring N1/C1-C4, Cg2 is the centroid of the pyrrole ring N2/C6-C9, Cg3 is the centroid of the pyridyl group N25-C24-C23-C22-C27-C26 and Cg4 is the centroid of the pyridyl ring N45A-C44A-C43A-C42A-C47A-C46A (Figure S13, Table S4).

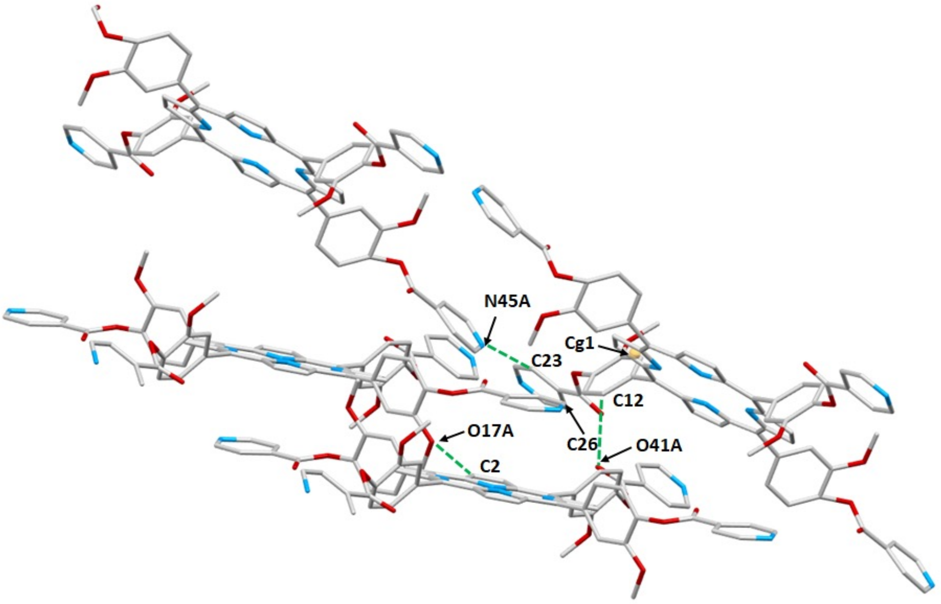

Figure S12. Drawing illustrating the Cg–H···O intermolecular interactions in compound **1**.

Table S3. Selected intermolecular interactions for compound **1**.

D –H···A[a]	Symmetry of A	D···A (Å)	D–H···A (°)
C2-H2…O17A	-1+x,y,z	3.433(5)	162
C12-H12…O41A	-x,-1/2+y,1/2-z	3.068(20)	113
C23-H23…N45A	1+x,-1+y,z	3.400(12)	143
C26-H26…Cg1	1+x,1/2-y,-1/2+z	3.669(7)	132

[a] : D = donor atom and A = acceptor atom.

Cg1 is the centroid of the N1/C1-C4 pyrrole ring.

Table S4. π-π Interactions (Å, °) in the crystal structure of compound **1**.

Cg(I)	*Cg(J)*	*Cg…Cg*	*α*	*CgI_Perp*	*CgJ_Per*	*Slippage*
Cg1	Cg3	3.835(3)	25.7	33.0	3.217(2)	3.456(2)
Cg2	Cg3	4.193(8)	35.2	13.2	2.800(2)	4.082(8)

Cg…Cg = distance between ring centroids, *α* = dihedral planes *I* and *J*, *CgI_Per* = perpendicular distance of *CgI* on ring *J*, *CgJ-Perp* = perpendicular distance of *CgI* on ring *I*.

Cg1 = centroid of the five membered ring N1/C1-C4; Cg2 = centroid of the five membered ring N2/C6-C9; Cg3 = centroid of the pyridyl ring N25-C24-C23-C22-C27-C26; Cg4 = centroid of the pyridyl ring N45A-C441-C43A-C42A-C47A-C4

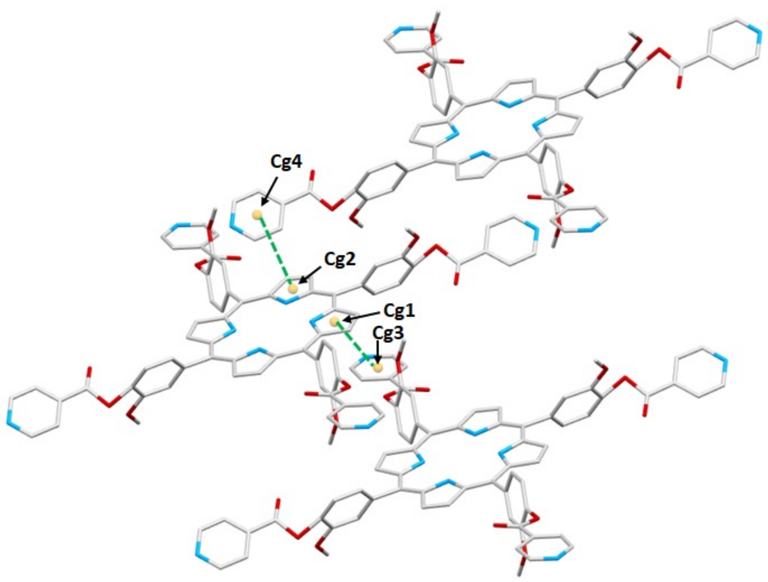

Figure S13. Drawing illustrating the Cg…Cg π intermolecular interactions in compound **1**.
